# Feasibility and implementation of a grocery shopping intervention for adults diagnosed with or at-risk for type 2 diabetes

**DOI:** 10.1017/S1368980023001453

**Published:** 2023-10

**Authors:** Kelseanna Hollis-Hansen, Sherey Tan, Sarah Bargnesi, Lily McGovern, Julia Drozdowsky, Leonard H Epstein, Lucia A Leone, Eunice Mak, Jaclyn Masci, Stephanie Anzman-Frasca

**Affiliations:** 1 Peter O’Donnell Jr. School of Public Health, UT Southwestern Medical Center, Dallas, TX, USA; 2 Harold C. Simmons Comprehensive Cancer Center, Dallas, TX, USA; 3 Department of Pediatrics, Jacobs School of Medicine and Biomedical Sciences, University at Buffalo, Buffalo, NY, USA; 4 Center for Ingestive Behavior Research, University at Buffalo, Buffalo, NY, USA; 5 Department of Community Health and Health Behavior, School of Public Health and Health Professions, University at Buffalo, Buffalo, NY, USA

**Keywords:** Default choices, Intervention, Grocery shopping, Implementation, Type 2 diabetes

## Abstract

**Objective:**

To examine the feasibility and implementation of an optimal defaults intervention designed to align grocery purchases with a diet recommended for people with or at-risk for type 2 diabetes.

**Design:**

This was a 5-week pilot randomised trial with three groups: in-person grocery shopping, shopping online and shopping online with ‘default’ carts. Participants were asked to shop normally in Week One, according to group assignment in Weeks Two–Four (intervention period), and as preferred in Week Five. All groups received diabetes-friendly recipes via email each intervention week.

**Setting:**

Participants grocery shopped in person or online. Grocery receipt forms, enrolment information and exit surveys were collected remotely and used to assess feasibility and implementation.

**Participants:**

Sixty-five adults with or at-risk for type 2 diabetes.

**Results:**

Sixty-two participants completed the exit survey and fifty-five submitted receipts all 5 weeks. Forty utilised recipes, 95 % of whom indicated recipes were somewhat or very useful. Orange chicken, quesadillas and pork with potato and apples were the most liked recipes. Most Defaults group participants accepted at least some default cart items. Recipes with the highest default acceptance were whole grain pasta and chicken, quesadillas with black beans and chicken with olives. Participants’ primary concerns about the intervention were costs associated with online shopping, inability to select preferred foods and some recipes including ingredients household members would not eat.

**Conclusions:**

The study had high retention, data were successfully collected remotely and the intervention was acceptable to most participants. Tailoring recipes to household preferences may be beneficial in future studies.

In recent estimates, 35 % of adults met criteria for pre-diabetes and 13 % have been diagnosed with type 2 diabetes in the USA^(1)^. In an observational study of US adults (*n* 9939), people living with diabetes had lower healthy eating index scores than people without diabetes^(2)^, a known risk factor for adverse metabolic and general health outcomes^(3)^. It has been suggested that nutrition interventions which result in improvements in diet quality and significant weight loss reduce diabetes-related health risks^(4)^ and engender remission for people with pre-diabetes and diabetes^(5)^. However, challenges remain in identifying clinically meaningful dietary interventions that are feasible, acceptable and implementable^(6)^. Grocery purchases and food selections precede consumption and are strongly correlated with diet quality^(7)^. By identifying strategies that make it easier to select and prepare recommended foods, we may be able to promote intake of meals that align with a diet for people with or at-risk for type 2 diabetes.

In a 2018 review, Jilcott Pitts *et al*. summarised that online grocery shopping may offer opportunities for improving food purchases through ‘reduced unhealthy impulse purchases’ and ‘nutrition labelling strategies’^(8)^. Conversely, online grocery shopping may have the unintended consequence of decreasing health-promoting purchases, given participants’ hesitance to rely on other people to select their produce and fears around losing money on a product they may not eat^(9,10)^. In an analysis of 137 shoppers from Maine, researchers found that online grocery shopping was associated with lower spending on candy and desserts than shopping in-person^(10)^, and in a study of 310 Maryland residents, participants reported purchasing sweets less frequently online than in-store^(11)^. However, in the latter study participants also reported purchasing fruits and vegetables less frequently online. Researchers have also raised concerns around retailers marketing ultra-processed sweet and savoury snacks online and how that might impact purchases^(12)^. The SARS-CoV-2 (COVID-19) pandemic had a tremendous influence on the online food retail market, with more retailers expanding to online platforms and increased retail sales and customers^(13)^. Current market research projections suggest that the number of online grocery users is expected to continue to increase year-over-year^(14)^. Overall, more research is needed on the potential of the online grocery shopping environment to promote healthy eating, with recent increases in online grocery shopping highlighting the timeliness of such questions.

Patients with or at-risk for diabetes are one population who may benefit from health promotion efforts in the context of online grocery shopping. Patients with diabetes have reported that ‘unhealthy food is hard to give up,’ ‘nutrition education is difficult to access’ and that ‘it is hard to know what food is healthy’^(15)^. Taken together, there is an opportunity to explore interventions that may promote diets recommended for people with or at-risk for type 2 diabetes in an online shopping environment, with initial emerging support for behavioural economic intervention approaches.

Nudges are one type of behavioural economic intervention that could be introduced in an online grocery shopping environment to promote healthier purchases. Gustafson *et al*. randomised 184 adults with no specific dietary needs to in-store grocery shopping, online shopping with no support and online shopping with nudges to encourage participants to shop online and purchase more fruits and vegetables (e.g. meal ideas, recipes, online support group). They found that those who shopped online and received nudges spent more on fruits and vegetables than participants in the in-store or online-only group, without spending more on their grocery bill overall^(16)^. Their team reported 70 % adherence across the 8-week study period and provided preliminary evidence that online shopping with nudges may improve food purchasing behaviours.

Another potential behavioural economic strategy is optimal defaults. Default options are pre-determined or automatic choices. Research has shown that optimal default options increase the likelihood of engaging in a target behaviour (e.g. organ donation, ordering healthier side dishes)^(17)^. In a meta-analysis comparing the impact of default options on numerous behaviours, optimal defaults were found to exert a stronger influence on consumer behaviours than pro-environmental behaviours, and defaults related to food choice were particularly promising^(17)^. Coffino *et al*. recently conducted three pilot studies that explored using prefilled, ‘default’ grocery carts to improve the nutritional quality of grocery purchases specifically^(18–20)^. In their first study, undergraduates that shopped with prefilled carts purchased more nutrient-dense foods and fewer calories than a nutrition education group in a hypothetical grocery purchasing task^(18)^. In two follow-up studies, people who used food pantries and lived in single-resident households were randomised to receive default grocery carts or nutrition education in a single session (study one)^(19)^ or for 5 weeks (study two)^(20)^. Researchers found the default intervention group purchased more nutrient-dense groceries than the nutrition education control in the single time-point study^(19)^ and over time in the 5-week study^(20)^. Given previous success using optimal defaults to improve consumer behaviour more broadly^(17)^ and specifically to improve grocery selections among undergraduates^(18)^ and people with financial constraints^(19,20)^, default options may also help other populations make healthier grocery purchases when shopping online.

Our study differs from the Coffino *et al*. and Gustafson *et al*. research described above in several ways that extend their findings. First, our study focused on a population with or at-risk for type 2 diabetes and promoted a diet recommended for this population, which broadens the use of defaults to potentially prevent and improve a disease state. To test this approach in the context of everyday shopping, participants in this study did not attend appointments in the laboratory, were not restricted on the amount they could spend or the number of people living in their home and were free to shop in their own environment at their preferred time during the week. Participants were randomised to one of three groups: (1) in-person grocery shopping (‘Control’, *n* 21), (2) online grocery shopping (‘Online’, *n* 21) and (3) online shopping with prefilled ‘default’ grocery carts (‘Defaults’, *n* 23). Participants shopped as usual in Week One of the study, per their group assignment in Weeks Two through Four, and however they preferred in Week Five. All participants received recipes tailored to a diet for people with or at-risk for type 2 diabetes, and the Defaults group had their online grocery carts prefilled with recipe ingredients during each week of the 3-week intervention period. Overall, participants in the Defaults group had improvements in the nutritional quality of grocery purchases compared to participants in the Control and Online groups^(21)^.

In this article, we discuss measures and outcomes specifically relating to the feasibility and implementation of this study. Implementation outcomes have been conceptualised and defined as acceptability (satisfaction with intervention), appropriateness (perceived usefulness), feasibility (practicability), adoption (uptake), cost, fidelity (adherence), penetration (level of spread) and sustainability (long-term durability)^(22)^. Previous studies on default interventions present some evidence in support of implementation success, such as high participant acceptance of default items and low dropout^(18–20)^. Given the emerging evidence supporting the promise of default interventions in the context of online grocery shopping, it is important to report additional data on feasibility and implementation to refine these interventions before implementing and evaluating them on a larger scale. Information, such as effective recruitment strategies, participants’ liking and perceived usefulness of study recipes, acceptance of default grocery cart items by recipe and willingness to use recipes and shop online in the future, could be beneficial to researchers interested in designing online grocery interventions, particularly for those that incorporate default options. Therefore, the goal of this article is to describe relevant components of implementation for this intervention and highlight next steps for subsequent studies to build upon these learnings.

## Methods

The methodology used in this article was informed by a feasibility study conducted by Di Noia *et al*.^(23)^, best practice guidelines for feasibility evaluation put forth by Bowen *et al*.^(24)^ and Conn *et al*.^(25)^ and implementation evaluation conceptualised by Proctor *et al*.^(22)^ To examine aspects of implementation, such as fidelity, acceptability, appropriateness and adoption^(22–25)^, we describe study recruitment and enrolment, protocol adherence and acceptance and adoption of intervention components (study recipes, online grocery shopping and default grocery carts).

Study enrolment data were collected from eligibility screeners which included items that indicated whether participants met the inclusion criteria as well as how the participant heard about the study. To measure enrolment feasibility, we calculated the proportion of eligible participants out of the total screened, the proportion of those eligible that enrolled and the proportion of enrolled participants recruited through each method to help researchers identify the best recruitment tactics for future studies and consider potential changes to some eligibility criteria^(24)^. As another feasibility measure, we also report on the proportion of participants whose healthcare providers provided diabetes diagnosis or risk data after receiving a Health Insurance Portability and Accountability Act (HIPAA) release and request to do so.

### Recruitment, eligibility screening and enrolment

#### Recruitment

Recruitment took place from Summer 2019 through Fall 2020 except for a brief period from March to May of 2020 when the study was paused due to the initial COVID-19 outbreak in the USA. Although grocery stores were designated an essential service and most remained open during US lockdowns, the research team had concerns over how food shortages, limited store hours and store closures may impact the grocery purchasing experience during this time. Eleven participants (17 %) were recruited and completed all study procedures in early 2020, and the remaining 54 (83 %) were enrolled after recruitment started again in May 2020.

Forty-six percentage of participants were recruited through social media advertisements. Social media advertisements were posted on Facebook once per month from June–October of 2020 and cost $360 USD total, with promotional costs ranging from $70 to $120 per post depending on level of engagement. Posts were also shared, but not promoted monetarily, through our laboratory’s Twitter account. Twenty-three percentage of participants were recruited through listservs or websites, including advertisements sent to email lists maintained by our division, the Clinical and Translational Sciences Institute Buffalo Research Registry, the university’s i2b2 listserv^(27)^ and postings on our laboratory website. Twelve percentage were recruited through ResearchMatch.org, 12 % through flier or newspaper advertisements and 6 % through other methods (e.g. event tabling).

#### Eligibility screening criteria

Eligibility criteria included (1) 18 years of age or older, (2) a self-reported diagnosis of pre-diabetes or type 2 diabetes or increased risk of developing type 2 diabetes determined by American Diabetes Association type 2 diabetes risk screening results (available at: www.diabetes.org/risk-test), (3) primary household grocery shopper, (4) at least 50 % of weekly grocery purchases made in-person at one of two of the largest grocery stores in the region in which the research was conducted, (5) willingness to grocery shop online if randomised to an online group, (6) English fluency, (7) no current participation in the Supplemental Nutrition Assistance Program (SNAP) or the Special Supplemental Nutrition Assistance Program for Women, Infants, and Children (WIC), (8) no dietary restrictions that limit consumption of study recipes (e.g. liquid diet, vegan diet, strict ketogenic diet) and (9) no recent participation in a grocery purchasing or eating behaviour study. At the time the study was designed, the online grocery platform used in this study was not accepting any food assistance benefits online; therefore, we excluded people receiving SNAP/WIC from this pilot as they might not have been able to shop online. We also excluded people that did not make purchases from the two largest retailers in the region as there were logistical challenges to populating the prefilled grocery carts with the same or similar default food options across multiple retailers each week.

#### Eligibility screening results

Of the 1144 people who completed the eligibility screener, 76 % were ineligible. Reasons for ineligibility were not reporting or meeting type 2 diabetes risk criteria (51 %), not shopping at study grocery stores (23 %), currently receiving SNAP or WIC benefits (10 %), not willing to shop online (7 %), not the primary grocery shopper (4 %), dietary/health restrictions that limited consumption of study foods (3 %) and other reasons (2 %).

#### Enrolment

Participants (*n* 65) were randomised to a study group using block randomisation with participants stratified by sex and diabetes status (diagnosis *v*. risk). Thirty-five percentage of the study sample reported a diagnosis of type 2 diabetes (*n* 23), and 65 % (*n* 42) reported pre-diabetes or risk. Eligible participants who proceeded to enrolment were asked to submit a HIPAA release, to allow staff to verify type 2 diabetes or diabetes risk status with their health care provider, though it was not required to participate. Consent forms and HIPAA release forms were primarily sent and returned by email but were also provided by paper mail if preferred by the participant. Thirty-one percentage of participants had their diabetes, pre-diabetes or risk status verified by a health provider (*n* 20).

#### Protocol adherence

**Fig. 1:**
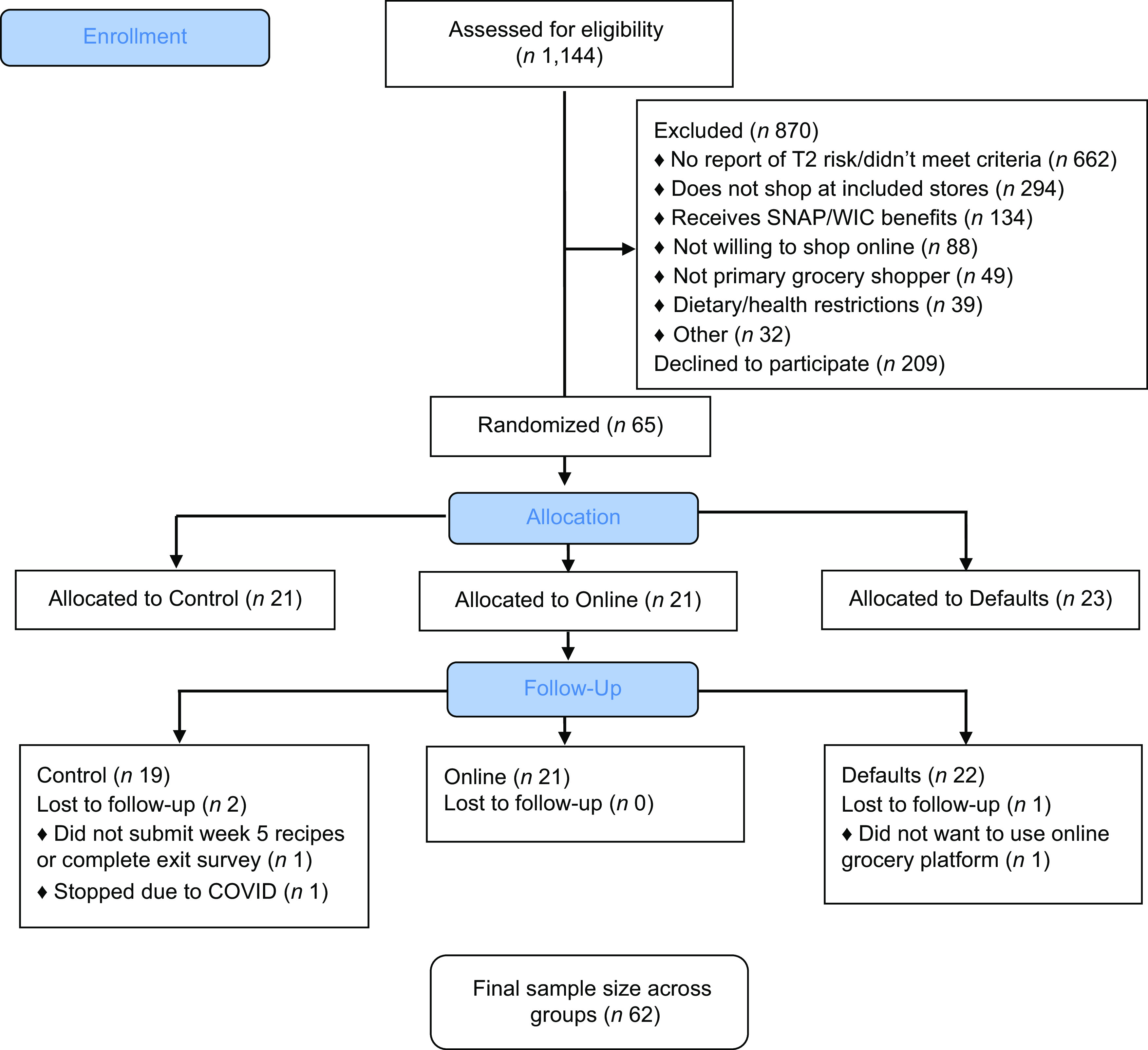
CONSORT flow diagram: implementation of behavioural grocery intervention

Complete protocol adherence was defined as submitting a baseline questionnaire, submitting grocery receipt data at least once per week for each of the 5 weeks of the study, and completing the exit survey. Sixty-five participants (100 %) submitted at least some receipt data after randomisation, 55 (85 %) submitted grocery receipt data during all 5 weeks of the study and 62 (95 %) completed the exit survey. All participants submitted Week One (baseline) receipt data, 64 submitted Week Two and Three receipts (99 %), 58 submitted Week Four receipts (89 %) and 62 submitted Week Five receipts (95 %). Of the fifty-five participants that submitted all grocery receipts, fifty-four also completed the exit survey; thus, 83 % of people had complete protocol adherence. See also Fig. 1 for a CONSORT diagram^(27)^ on study recruitment, exclusion, randomisation and follow-up.

### Measures

#### Baseline questionnaire

After consent, baseline questionnaires were emailed to participants. The questionnaire included demographics (age, race/ethnicity, height/weight, sex, education), how often participants usually grocery shopped (bi-weekly, weekly, or more than once per week) and where participants shopped outside of the study stores, including other grocers, farmers’ markets, corner stores, restaurants and coffee shops to better understand usual food purchasing behaviour.

#### Food receipt measures

Throughout the study, participants submitted store receipts and food receipt forms adapted from prior grocery studies^(7)^. Food receipt forms were used to gather additional details on food and beverage items purchased (brand, size and quantity), the store where items were purchased and how the items were purchased (online *v*. in-store), as these details are not consistently provided on store receipts. Participants completed the forms at home each week (One–Five) and returned them by email. Staff called participants to review food receipt forms, ask clarification questions if needed and confirm information was accurate and complete.

#### Exit survey

At the end of the study, participants completed an online exit survey which asked participants to provide feedback on study recipes, online shopping during the study if they indicated they had shopped online during the study period, default shopping carts if they were in the default shopping group, plans to grocery shop online in the future and what they would change about the study.

#### Recipes

Research staff adapted publicly available DASH diet recipes for this study (e.g. recipes from Mayo Clinic, EatingWell, etc.), which were then edited by a registered dietitian (author JM) to ensure alignment with recommendations for people with or at-risk for type 2 diabetes. Recipes were designed to be applicable to all participants regardless of gender, activity level and disease severity. For this reason, we chose not to adhere to rigid guidelines for calories or specific nutrients, rather we had target ranges for calories, Na, carbohydrates and saturated fat. We aimed for the following parameters for each serving: 45–60 g of carbohydrates, 400–500 calories, variety and balance of food groups (whole grains, fruits, non-starchy vegetables, lean proteins, low-fat dairy), adequate fibre and moderate or low in saturated fat and Na.

While people with dietary restrictions that could not be accommodated were excluded, recipes were adapted to meet some specific needs. Eighty-five percentage of participants received the standard Western diet recipes, with the remaining participants receiving gluten-free (6 %, *n* 4) or vegetarian (6 %, *n* 4) recipes. Alternative vegetarian and gluten-free recipes were added to the study recipe bank prior to the start of the study. Participants who needed further alterations received recipes from the study recipe bank with some ingredients modified to accommodate the need (e.g. kosher adaptations, 3 %, *n* 2). All alternative recipes met the same target criteria and included the same number of DASH default items as the standard recipes. All recipes were standardised to four servings.

#### Recipe measures

To examine recipe acceptability, appropriateness and adoption, we used responses collected from the exit survey to describe whether participants used study recipes (yes/no), how many found the recipes useful (not useful at all, somewhat useful, very useful) and which recipes were the most used and liked. Response options included: I did not receive this recipe, I did not make this recipe, I did not like at all, I liked a little, I liked somewhat and I liked a lot. We also evaluated willingness to use recipes in the future (yes/no). Measures were calculated for all participants and by study group.

#### Online grocery shopping measures

To assess online grocery shopping fidelity food receipt forms were coded to determine how many of the intervention weeks participants in the Online and Defaults groups used the online grocery platform as requested. To measure acceptability, participants rated their online shopping experience as Excellent, Good, OK, or Poor in the exit survey. We also evaluated willingness to use online grocery shopping carts in the future (yes/no).

#### Defaults group measures

Research staff and participants both had access to the participants’ online grocery shopping accounts. Participants’ financial information was encrypted and inaccessible to research staff, but they had access to all other functionality and would use the shared account to prepopulate a grocery cart with default items that aligned with the DASH diet and study recipes for each week. Four to nine default ingredients were added to the participants’ carts each week.

For a measure of intervention integrity, participants in the Defaults group were asked if they saw the prefilled carts in the online grocery shopping platform (yes/no) in the exit survey. To measure intervention acceptance and appropriateness, exit surveys included an item on whether Defaults participants found the prefilled carts to be useful (not useful at all, somewhat useful, very useful). Research assistants independently double-coded the food receipt data to determine if the Defaults group purchased none (0), some (1) or all (2) of the default items placed into their online shopping carts. For an item to be considered an accepted default, the item had to be accepted exactly as is without modification. For example, if a participant substituted a 16-oz orange juice for a 64-oz orange juice, it was not considered an acceptance of the default, given the participant would have made a modification. Once research assistants coded the receipts, discrepancies were reconciled by a third reviewer, and proportions were calculated to determine the percentage of the sample purchasing none, some or all default items placed into the online shopping carts by week and by recipe. We also evaluated willingness to use prefilled grocery shopping carts in the future (yes/no).

#### Open-ended exit survey items

There were four open-ended response items in the exit survey; participants were not required to answer these items. The questions and response rates were: (1) After participants rated their online shopping experience, they were asked ‘Please tell us why you chose the response that you did’ (*n* 43, out of 43 that shopped online during the study period), (2) After participants rated recipe usefulness, they were asked ‘Please tell us why you chose the response that you did’ (*n* 39, out of 40 that reported using recipes), (3) ‘Please share any other comments that you have about the recipes [what you liked or didn’t like about them, if they were too easy/difficult, or any other thoughts you’d like to share]’ (*n* 33, out of 40 that reported using recipes) and (4) ‘What would you change about this study in the future to make it better for participants like you?’ (*n* 58, out of 62 respondents).

### Data analysis

Descriptive statistics were calculated using frequencies and proportions for categorical variables. Fisher’s exact tests were used to identify if there were group differences in recipe use, recipe usefulness, willingness to use recipes and online grocery shopping in the future. Inductive thematic analysis (familiarisation, coding, defining themes) was used to summarise feedback from open-ended survey responses regarding recipes, online grocery shopping and what participants would change about the study in the future to contextualise quantitative results.

### Study payment

Participants were paid up to $75 using a reloadable debit card as they completed study tasks: $20 after completing baseline measures, $10 after each week of intervention measures and another $25 for completing the exit survey.

## Results

### Participant characteristics

Among the sixty-five randomised participants in the analytic sample, participants were predominantly female (85 %), college graduates (70 %) and white (92 %). Annual household income varied, with 20 % below $50 000 USD, 47 % between $50 000 and$74 999 USD, 28 % above $100 000 USD and 5 % choosing not to say. Thirty-one percentage of participants reported having at least one child under 18 in the household and 75 % had at least one other adult in the home for an average household size of 2·5 people ± 1·3. Participants’ mean age was 53·2 years old ± 10·7, and mean BMI (calculated using participants’ self-reported height and weight) was 36·5 ± 7·8. We did not ask participants if they typically grocery shopped online in the baseline questionnaire, given that doing most of one’s grocery shopping in-person at the time of screening was an inclusion criterion, but did note that three participants (5 %) submitted online grocery receipts in the Week One (baseline) receipt collection. More information about baseline food purchasing behaviour can be found in Table 1.

**Table 1:** Food purchasing behaviour from baseline questionnaire

Grocery shopping frequency	(*n* 64)	%
Every other week	5	8
Every week	30	47
More than once per week	29	45

Food acquisition by other type of food retailer	(*n* 65)	%
Corner store	12	19
Farmers market	23	35
Fast food or quick service restaurant	38	59
Coffee shop	29	45

Values in columns are *n* – the subsample and % – the proportion of participants that indicated each response.

### Recipe results: fidelity, acceptability, appropriateness, adoption

Recipe use differed by group, such that participants in the Defaults group were more likely to report using study recipes (86 %) than participants in the Control (47 %) and Online groups (57 %), Fisher’s *P* = 0·02. Table 2 compares recipe use and usefulness by group. Among participants that used recipes during the study period, 90 % indicated they planned to use recipes again in the future. There were no group differences regarding willingness to use recipes in the future (Table 2). Of the standard recipes, the top three most liked were roasted orange chicken, chicken quesadillas with black beans and pork tenderloin with potato and apples. The three least likely to be made or liked included lentil stew, lentil medley and Mediterranean chicken. Table 3 describes participant use and liking of each recipe.

**Table 2:** Reported use and usefulness of study recipes via online exit survey post-intervention (*n* 62)

	Across groups (*n* 62)				Control (*n* 19)				Online (*n* 21)				Defaults (*n* 22)				
	Yes		No		Yes		No		Yes		No		Yes		No		Fisher’s
	*n*	%	*n*	%	*n*	%	*n*	%	*n*	%	*n*	%	*n*	%	*n*	%	*P*
Used recipes	40	64·5	22	35·5	9	47·4	10	52·6	12	57·1	9	42·9	19	86·4	3	13·6	*P* = 0·02

	Across groups (*n* 40)						Control (*n* 9)						Online (*n* 12)						Defaults (*n* 19)						
	Not at all		Somewhat		Very		Not at all		Somewhat		Very		Not at all		Somewhat		Very		Not at all		Somewhat		Very		Fisher’s
	*n*	%	*n*	%	*n*	%	*n*	%	*n*	%	*n*	%	*n*	%	*n*	%	*n*	%	*n*	%	*n*	%	*n*	%	*P*
Recipes Useful*	2	5	27	67·5	11	27·5	0	0	6	66·7	3	33·3	0	0	8	67·7	4	33·3	2	10·5	13	68·4	4	21·1	*P* = 0·78

	Across groups (*n* 40)				Control (*n* 9)				Online (*n* 12)				Defaults (*n* 19)				
	Yes		No		Yes		No		Yes		No		Yes		No		Fisher’s
	*n*	%	*n*	%	*n*	%	*n*	%	*n*	%	*n*	%	*n*	%	*n*	%	
Plan to use recipes in future*	36	90	4	10	9	100	0	0	10	83·3	2	16·7	17	89·5	2	10·5	*P* = 0·67

Fisher’s = Fisher’s exact test, a chi-square variation that accounts for small cell sizes.

* These questions were asked among the forty participants who used study recipes. Values in columns are *n* (%) – the subsample (*n*) and proportion (%) of participants that indicated each response.

**Table 3:** Participants use and liking of study recipes by recipe (*n* 40)

	Did not receive		Did not make		Did not like		Liked a little		Like somewhat		Liked a lot	
	*n*	%	*n*	%	*n*	%	*n*	%	*n*	%	*n*	%
Pasta and grilled chicken ^**W**^	1	2·5	16	40	2	5	1	2·5	10	25	10	25
Orange chicken ^**W/GF/K**^	0	0	6	15	1	2·5	3	7·5	11	27·5	19	47·5
Vegetable lentil stew ^**W/GF/K/V**^	1	2·5	17	42·5	1	2·5	4	10	7	17·5	10	25
Chicken quesadilla ^**W/GF**^	3	7·5	12	30	0	0	0	0	9	22·5	16	40
Grilled chicken and olives ^**W/GF/K**^	4	10	20	50	1	2·5	1	2·5	4	10	10	25
Taco salad ^**W/GF/K/V/**^ *	3	7·7	14	35·9	0	0	1	2·6	6	15·4	15	38·5
Mediterranean chicken ^**W/GF**^	2	5	22	55	2	5	3	7·5	5	12·5	6	15
Lentil medley ^**W/GF/K/V**^	3	7·5	26	65	1	2·5	4	10	4	10	2	5
Pork, potatoes and apples ^**W/GF/**^ *	2	5·1	15	38·5	1	2·6	0	0	4	10·3	17	43·6

W, Western; GF, gluten-free; K, Kosher; V, vegetarian.

Values in columns are *n* (%) – the subsample (*n*) and proportion (%) of participants that indicated each response.

* *n* 39, participant missed item.

The most frequent positive feedback on study recipes was that they were easy to follow. Across all groups, participants received three recipes per week, and some articulated a desire to receive more. Some participants expressed specific recipes and food items (e.g. lentils, quinoa) did not fit with their preferences or their family’s preferences which limited usefulness of those recipes, ‘*Some were tasty. Some were things that I would not eat or be able to convince anyone in my house to eat, Lentil Stew, for example*’. This feedback aligns with descriptive statistics that suggest lentil and quinoa dishes were the least likely to be cooked and enjoyed by participants.

### Online grocery shopping results: fidelity, acceptability, appropriateness, adoption

Of the participants that reported grocery shopping online during the study period (*n* 43), most rated the experience as excellent (*n* 12, 28 %) or good (*n* 16, 37 %), some rated it as OK (*n* 12, 28 %) and three rated the experience as poor (7 %). A majority of Online and Defaults participants randomised to grocery shop online during the study period adhered to those instructions for all or most of their shopping (Table 4). In the follow-up survey, two Control participants reported grocery shopping online at least once, which was confirmed by their food receipts. Of the forty-three participants that reported grocery shopping online, 28 % (*n* 12) indicated they will not grocery shop online again, 70 % (*n* 30) indicated they will do some grocery shopping online and one person indicated they will do all grocery shopping online in the future. There were no group differences on willingness to use online grocery shopping in the future (Table 4).

**Table 4:** Participants’ in-person and online grocery shopping by group

	Control						Online						Defaults					
	In-person		Online		Both		In-person		Online		Both		In-person		Online		Both	
	*n*	%	*n*	%	*n*	%	*n*	%	*n*	%	*n*	%	*n*	%	*n*	%	*n*	%
Week 2 (*n* 63)	21	100	0	0	0	0	2	10	12	60	6	30	2	9	14	64	6	27
Week 3 (*n* 63)	18	90	2	10	0	0	1	5	15	71	5	24	2	9	13	59	7	32
Week 4 (*n* 57)	18	100	0	0	0	0	1	5	12	63	6	32	2	10	13	65	5	25

	Control (*n* 2)						Online (*n* 20)						Defaults (*n* 21)					
	Always		Sometimes		No		Always		Sometimes		No		Always		Sometimes		No	
	*n*	%	*n*	%	*n*	%	*n*	%	*n*	%	*n*	%	*n*	%	*n*	%	*n*	%
Plan to grocery shop online in future (*n* 43)	0	0	2	100	0	0	0	0	13	65	7	35	1	4·8	15	71·4	5	23·8

Values in columns are *n* (%) – the subsample (*n*) and proportion (%) of participants that indicated each response. 1 participant in the In-person group and 1 participant in the Online group did not submit Week 2 receipts; 2 participants in the Online group and 2 participants in the Defaults group did not submit Week 4 receipts.

#### Barriers of online shopping

In the primary outcomes paper for the overarching study, the research team reported that average dollars spent at study grocery stores did not differ between group^(21)^. However, multiple participants perceived online shopping to be more expensive than shopping in-person (*n* 10 out of the 43 that responded to the open-ended question about online grocery shopping), and a few indicated the inability to use coupons as a barrier (*n* 7). Five participants out of the forty-three that responded to the open-ended question about online grocery shopping (12 %) mentioned that they prefer to pick out their own groceries and felt there was too much variability in the quality of produce selected by shoppers working for the online grocery platform.

#### Benefits of online shopping

The most frequently cited benefits of online grocery shopping were time saved and convenience. One participant wrote ‘*Ordering online and picking up the groceries was so much easier on me. I don’t have to deal with overcrowded grocery stores and the stress of not being able to find what I need in the store*’. And another said, ‘*I was hesitant at first to order produce online, but when I did, the produce chosen was in very good shape. It was pretty much what I would have chosen in the store. I really loved that it saved me so much time*’.

### Defaults results: fidelity, acceptability, appropriateness, adoption

Among Defaults participants that completed an exit survey, all twenty-one indicated they saw the prefilled grocery cart while shopping (100 %). The recipes with the highest default acceptance were pasta and grilled chicken (81 %), chicken quesadillas with black beans (84 %) and grilled chicken with olives (90 %). Table 5 details default acceptance by recipe and study week. Most Defaults participants found the prefilled carts to be somewhat or very useful: 19 % (*n* 4) did not find the prefilled carts to be useful at all, 62 % (*n* 13) found the carts to be somewhat useful and 19 % (*n* 4) found the carts to be very useful. Seventy-six percentage (*n* 16) indicated that they plan to buy default items in the future.

**Table 5:** Defaults group acceptance of default items by recipe and week

	Received recipe		No defaults accepted		Some defaults accepted	
Acceptance by recipe	*n*		*n*	%	*n*	%
Standard western recipes						
Pasta and grilled chicken ^W^	16		3	19	13	81
Orange chicken ^W/GF/K^	20		6	30	14	70
Vegetable lentil stew ^W/GF/K/V^	22		5	23	17	77
Chicken quesadilla ^W/GF^	19		3	16	16	84
Grilled chicken and olives ^W/GF/K^	20		2	10	18	90
Taco salad ^W/GF/K/V^	22		5	23	17	77
Mediterranean chicken ^W/GF/K^ *	19		5	26	14	74
Lentil medley ^W/GF/K^ * ^/V^	21		5	24	16	76
Pork, potatoes and apples ^W/GF^	19		6	32	13	68
Alternative recipes						
Paella ^GF^	3		0	0	3	100
Polenta ^K/V^	3		2	70	1	30
Veggie kebabs ^F/V^	3		1	30	2	70
Quinoa and arugula ^V^	2		1	50	1	50
Broccoli garlic Pasta ^V^	2		1	50	1	50
Pasta primavera ^V^	2		0	0	2	100
Summer salad ^V^	2		0	0	2	100

	No receipt submitted		None		Some	
Acceptance by week	*n*	%	*n*	%	*n*	%
Week 2	0	0	1	5	21	95
Week 3	0	0	2	9	20	91
Week 4	1	5	3	14	18	82

W, Western; GF, gluten-free; K, Kosher; V, vegetarian.

Values in columns are *n* (%) – the subsample (*n*) and proportion (%) of participants that indicated each response.

* Kosher participant would have received these recipes but skipped Week 4; no participants accepted all default items.

### What participants would change about the study

The most frequently recommended change was an amendment to the food receipt form to make it quicker and easier to detail and submit information on grocery purchases. Multiple participants indicated that the forms were not ‘user-friendly’. The second most frequently mentioned change was providing additional recipes to allow more flexibility and choice in the recipes and default items received.

## Discussion

Findings from this study suggest that it was feasible to implement an optimal defaults intervention to improve the nutritional quality of grocery purchases for people with or at-risk for diabetes^(21)^ and collect all study data remotely. Most participants found the methods, data collection and prefilled default grocery carts to be acceptable and useful. Opportunities to further improve participant satisfaction and compliance include providing a greater variety of recipes and default items and/or tailoring recipes and default items to participant and household preferences as well as making improvements to the food receipt forms and/or using a different data collection format for grocery purchasing data.

Fifty-five (83 %) of our participants had complete protocol adherence, including submitting receipts across all study weeks. A strength of this study is the high rate of participant compliance and retention, though findings should be replicated in longer, larger studies. A systematic review of 174 longitudinal cohort studies reported that ‘flexibility in data collection methods might be most effective in maximizing retention’^(28)^. Allowing participants to consent, shop and submit data remotely by email and/or telephone calls may have encouraged compliance and retention and may be a way for researchers to deliver grocery interventions in the future. Another strength is that this study is consistent with other researchers’ findings that suggest providing behavioural economic interventions (e.g. nudges, defaults) through an online grocery shopping platform is feasible and may improve the nutritional quality of purchases^(16,18–20)^ and extends these findings to support a population with or at-risk for type 2 diabetes.

A limitation of this study is the homogeneity of the sample, which is predominantly white and college educated. There are several ways to open eligibility criteria in the future to improve heterogeneity. Given that 25 % of people that completed the eligibility screener were excluded for not shopping at one of the study stores, future research could modify or extend the stores that are included, with an eye towards stores in under-resourced neighbourhoods. In the present study, two of the largest grocery chains in the researchers’ geographic area were selected as study stores, to broaden external validity to the extent possible for a pilot study. That decision also helped with the feasibility of filling default grocery shopping carts. Since the design of this study, additional grocery stores are available on the online grocery platform used in this study, offering the potential to include other study stores in the future.

In addition, 134 people that completed the eligibility screener were excluded for receiving SNAP/WIC benefits. At the time this study was designed, the grocery platform used for this study did not accept electronic benefits as a form of payment and therefore the research team did not want to recruit participants that may not be able to shop online. Since then, SNAP purchasing pilots have expanded, and many retailers are approved to accept SNAP online^(29)^. Limits remain for WIC redemption, though the agency supports the transition and has a pilot underway to inform online expansion^(30)^. While the current findings have limited generalisability to people with lower-income or people receiving SNAP/WIC, previous research specifically recruited food pantry clients and found prefilled grocery carts to be an effective intervention for improving the nutritional quality of participants’ purchases during a single laboratory visit^(19)^ and over time^(20)^. Future research should aim to recruit a more heterogeneous sample, including people from under-resourced communities, people receiving SNAP and people from racially and ethnically minoritised groups to augment our understanding of whether online grocery shopping interventions are desirable and equitable^(9)^.

Food purchases are an antecedent to food consumption and are strongly correlated with diet quality^(7)^. However, another limitation of this study is that food purchases were the only outcome measured over time. In the overarching study, researchers calculated nutritional quality scores to confirm whether participants’ grocery purchases were aligned with recommendations for people with or at-risk for diabetes, and results suggested the Defaults intervention improved the nutritional quality scores based on the receipt data submitted^(21)^. However, receipts were not collected for all food purchases (e.g. purchases made at restaurants, corner stores, farmers markets’) and we did not measure actual intake objectively verified with laboratory values (e.g. blood glucose). Future research could collect additional purchasing and intake data and measure laboratory values that would confirm participants are consuming food they purchase during the study and improving health outcomes. Better understanding aspects of implementation, such as default acceptance and study feasaibility, was an exploratory aim reported on in this article. In the future, researchers could design their studies to comprehensively assess aspects of implementation from the outset of a study.

About 65 % of participants used study recipes, with greater usage in the Defaults group, in which 86 % reported using recipes. Nutrition and cooking education is a widely used strategy to improve dietary outcomes for people with or at-risk for diabetes and one of the best known examples is the diabetes prevention program^(31)^. Researchers have suggested that these strategies may be more effective when paired with behavioural interventions or embedded within a multi-component strategy, than when implemented as a standalone intervention^(32,33)^. In our study, we found that nudging participants to purchase specific foods via the default carts also encouraged them to use the healthier recipes that provided instructions on how to prepare those ingredients. This is one example of how behavioural economic interventions may work synergistically with established cooking and nutrition education programs like the diabetes prevention program.

Most participants in the Defaults group accepted at least one of the ingredients in the default carts each week (82–95 %) which also suggests that the intervention may be acceptable to participants. Out of the standard recipes, acceptance was highest for whole grain pasta and grilled chicken, chicken quesadillas and grilled chicken and olives. Researchers interested in conducting a similar study could use the study recipes most liked by participants (Table 2) with the highest default acceptance (Table 4) and can refer to the primary outcome paper to access a detailed description of recipe ingredients^(21)^. That said, while defaults are meant to make it easier for participants to eat more of the nutritious foods they may not already be eating, future studies could also consider innovative ways to provide recipes and populate grocery carts with items that may be more appetising or familiar to participants. One participant suggested ‘*offering a menu of recipes and choices*’ which provides the opportunity for customisation. Instead of assigning the same recipes to everyone, participants could rate a variety of recipes on liking and willingness to consume, and the recipes they rate the highest could be used to populate their default carts.

Most participants in the Online and Defaults groups were compliant and shopped online, though some continued to supplement online shopping with in-person shopping, and not all participants liked shopping online. One participant dropped out of the study because they did not want to shop online, and of those who shopped online during the study, 28 % indicated they did not plan to shop online in the future. While online grocery shopping is expected to continue to grow^(14)^ and most participants indicated they would do at least some of their shopping online, not everyone wants to shop using those platforms and previous research suggests this may be particularly true for people with lower income who have heightened concerns around cost and food waste^(9–11)^. Therefore, researchers could also consider options for adapting prefilled grocery carts for in-store shoppers. Although some shoppers want to go to a physical store to inspect, select and purchase foods, they may still be willing to use a mobile application or website that prepopulates recipes and grocery lists with default items and could use that tool to guide their shopping. Previous qualitative studies found that mothers with low income already search online for new recipes and are open to using this technology, especially if it allows for customisation^(34,35)^.

The most frequent complaint from participants was challenges with the food receipt form, the tool used to collect food purchasing details that may not be included on a receipt (e.g. product weight, size, quantity or brand). Potential solutions could include partnering with an online platform to see the exact product(s) participants purchased or using food tracking or grocery shopping applications that allow participants to scan barcodes and create food diaries or shopping lists from their purchases, which may be more user-friendly. This study highlights the need for technological and practical advancements that improve the process of collecting purchasing data from participants and reduce the time it takes for study staff to conduct nutritional analysis of receipt data, not just for studies of grocery defaults, but to benefit the broader food purchasing literature.

In this pilot randomised controlled trial, we found that an optimal defaults intervention aiming to improve grocery purchases using prefilled default grocery carts is an acceptable, feasible and desirable intervention for many people with or at-risk for type 2 diabetes. Using the lessons learned from this study, extension of this research may include expanding inclusion criteria to recruit a more heterogeneous sample, providing participants with more recipe options, prepopulating default carts with items that may be more desirable to participants and members of their household and streamlining the process for collecting detailed receipt data. Future research could increase the study period beyond 5 weeks to identify whether compliance and retention remain high over longer periods of time and whether long-term dietary change is possible. Implementing recommendations identified in this pilot study may increase the likelihood of continued adherence to the study protocol and defaults recommendations.

## References

[1] CDC (2020) National Diabetes Statistics Report. https://www.cdc.gov/diabetes/pdfs/data/statistics/national-diabetes-statistics-report.pdf (accessed June 2022).

[2] McClure ST , Schlechter H , Oh S et al. (2020) Dietary intake of adults with and without diabetes: results from NHANES 2013–2016. BMJ Open Diabetes Res Care 8, e001681. doi: 10.1136/bmjdrc-2020-001681. PMC7590352 33099509

[3] Sanjeevi N & Freeland-Graves JH (2023) Low diet quality is associated with adverse levels of metabolic health markers and clustering of risk factors in adults with type 2 diabetes. J Hum Nutr Diet 36, 31–39. doi: 10.1111/jhn.13020. 35442546

[4] Fung TT , Li Y , Bhupathiraju SN et al. (2021) Higher global diet quality score is inversely associated with risk of type 2 diabetes in US women. J Nutr 151, 168S–175S. doi: 10.1093/jn/nxab195. 34689196 PMC8542093

[5] Taylor R , Ramachandran A , Yancy WS Jr et al. (2021) Nutritional basis of type 2 diabetes remission. BMJ 374, n1449. doi: 10.1136/bmj.n1449. 34233884 PMC8261662

[6] Franz MJ , Boucher JL , Rutten-Ramos S et al. (2015) Lifestyle weight-loss intervention outcomes in overweight and obese adults with type 2 diabetes: a systematic review and meta-analysis of randomized clinical trials. J Acad Nutr Diet 115, 1447–1463. doi: 10.1016/j.jand.2015.02.031. 25935570

[7] Appelhans BM , French SA , Tangney CC et al. (2017) To what extent do food purchases reflect shoppers’ diet quality and nutrient intake? Int J Behav Nutr Phy 14, 1–10. https://www.iriworldwide.com/en-us/forrester-online-grocery-snapshot 10.1186/s12966-017-0502-2 PMC5387266 28399887

[8] Jilcott Pitts SB , Ng SW , Blitstein JL et al. (2018) Online grocery shopping: promise and pitfalls for healthier food and beverage purchases. Public Health Nutr 21, 3360–3376. doi: 10.1017/S1368980018002409. 30338751 PMC10260851

[9] Trude ACB , Lowery CM , Ali SH et al. (2022) An equity-oriented systematic review of online grocery shopping among low-income populations: implications for policy and research. Nutr Rev 80, 1294–1310. doi: 10.1093/nutrit/nuab122. 35076065 PMC8990744

[10] Zatz LY , Moran AJ , Franckle RL et al. (2021) Comparing shopper characteristics by online grocery ordering use among households in low-income communities in Maine. Public Health Nutr 24, 5127–5132. 34030759 10.1017/S1368980021002238 PMC11082826

[11] Trude ACB , Ali SH , Lowery CM et al. (2022) A click too far from fresh foods: a mixed methods comparison of online and in-store grocery behaviors among low-income households. Appetite 175, 106038. doi: 10.1016/j.appet.2022.106038. 35421540

[12] Boyland E , Thivel D , Mazur A et al. (2020) Digital food marketing to young people: a substantial public health challenge. Ann Nutr Metab 76, 6–9. doi: 10.1159/000506413. 32101856

[13] Shen H , Namdarpour F & Lin J (2022) Investigation of online grocery shopping and delivery preference before, during, and after COVID-19. Transp Res Interdis Perspec 14, 100580. doi: 10.1016/j.trip.2022.100580. PMC8913267 35291491

[14] Kodali S , Swerdlow F & Hansen T (2022) US Online Grocery Snapshot: Q4 2021 IRI Data Shows Online Grocery Trends Emerging Post-Pandemic. https://www.forrester.com/report/us-online-grocery-snapshot-q4-2021/RES177029?ref_search=3471426_1644002555548&utm_content=EP%2Fth%2Fecommerce-reviews%2Fyotpo-reviews-and-pricing (accessed February 2022).

[15] Bross R , Genter P , Lu Y et al. (2022) Barriers to healthy eating and diabetes diet education: divergent perspectives of patients and their providers. (published correction appears in Health Educ Behav 2022; doi: 10901981221081528). Health Educ Behav 49, 658–666. doi: 10.1177/10901981211052241. 34713743

[16] Gustafson A , Gillespie R , DeWitt E et al. (2022) Online pilot grocery intervention among rural and urban residents aimed to improve purchasing habits. Int J Environ Res Public Health 19, 871. doi: 10.3390/ijerph19020871. 35055688 PMC8775883

[17] Jachimowicz JM , Duncan S , Weber EU et al. (2019) When and why defaults influence decisions: a meta-analysis of default effects. Behav Public Policy 3, 159–186. doi: 10.1017/bpp.2018.43.

[18] Coffino JA & Hormes JM (2018) A default option to enhance nutrition within financial constraints: a randomized, controlled proof-of-principle trial. Obesity 26, 961–967. doi: 10.1002/oby.22151. 29604181 PMC5970034

[19] Coffino JA , Udo T & Hormes JM (2020) Nudging while online grocery shopping: a randomized feasibility trial to enhance nutrition in individuals with food insecurity. Appetite 152, 104714. doi: 10.1016/j.appet.2020.104714. 32304731 PMC7482976

[20] Coffino JA , Han GT , Evans EW et al. (2021) A default option to improve nutrition for adults with low income using a prefilled online grocery shopping cart. J Nutr Educ Behav 53, 759–769. doi: 10.1016/j.jneb.2021.06.011. 34509276

[21] Anzman-Frasca S , McGovern L , Ferrante MJ et al. (2023) Effects of a grocery shopping intervention designed to improve diet adherence in diabetes: a randomized trial. Obesity (Silver Spring) 31, 62–73. doi: 10.1002/oby.23588. 36444835

[22] Proctor E , Silmere H , Raghavan R et al. (2011) Outcomes for implementation research: conceptual distinctions, measurement challenges, and research agenda. Adm Policy Ment Health 38, 65–76. doi: 10.1007/s10488-010-0319-7. 20957426 PMC3068522

[23] Di Noia J , Monica D , Sikorskii A et al. (2019) Feasibility of a farm-to-WIC intervention. Public Health Nutr 22, 3405–3415. 31405392 10.1017/S1368980019001976 PMC10260496

[24] Bowen DJ , Kreuter M , Spring B et al. (2009) How we design feasibility studies. Am J Prev Med 36, 452–457. doi: 10.1016/j.amepre.2009.02.002. 19362699 PMC2859314

[25] Conn VS , Algase DL , Rawl SM et al. (2010) Publishing pilot intervention work. West J Nurs Res 32, 994–1010. doi: 10.1177/0193945910367229. 20702685

[26] Bucalo M , Gabetta M , Chiudinelli L et al. (2021) i2b2 to optimize patients enrollment. Stud Health Technol Inform 281, 506–507. doi: 10.3233/SHTI210217. 34042623

[27] Moher D , Jones A , Lepage L et al. (2001) Use of the CONSORT statement and quality of reports of randomized trials: a comparative before-and-after evaluation. JAMA 285, 1992–1995. doi: 10.1001/jama.285.15.1992. 11308436

[28] Teague S , Youssef GJ , Macdonald JA et al. (2018) Retention strategies in longitudinal cohort studies: a systematic review and meta-analysis. BMC Med Res Methodol 18, 151. doi: 10.1186/s12874-018-0586-7. 30477443 PMC6258319

[29] USDA (2022) Stores Accepting SNAP Online. https://www.fns.usda.gov/snap/online-purchasing-pilot (accessed June 2022).

[30] USDA (2021) WIC Supports Online Ordering and Transactions in WIC. 11/2/2021. https://www.fns.usda.gov/wic/supports-online-ordering-transactions (accessed June 2022).

[31] Golovaty I , Ritchie ND , Tuomilehto J et al. (2022) Two decades of diabetes prevention efforts: a call to innovate and revitalize our approach to lifestyle change. Diabetes Res Clin Pract. Published online: 2 December 2022. doi: 10.1016/j.diabres.2022.110195. PMC10079599 36470316

[32] Perez-Cueto FJA (2019) An umbrella review of systematic reviews on food choice and nutrition published between 2017 and 2019. Nutrients 11, 2398. doi: 10.3390/nu11102398. PMC6836087 31591373

[33] Kullgren JT , Hafez D , Fedewa A et al. (2017) A scoping review of behavioral economic interventions for prevention and treatment of type 2 diabetes mellitus. Curr Diab Rep 17, 73. doi: 10.1007/s11892-017-0894-z. 28755061 PMC5619648

[34] Tobey LN , Mouzong C , Angulo JS et al. (2019) How low-income mothers select and adapt recipes and implications for promoting healthy recipes online. Nutrients 11, 339. 10.3390/nu11020339. 30764537 PMC6412388

[35] Leone L , Haynes-Maslow L , Kasprzak C et al. (2022) The WIC shopping experience: a qualitative study examining retail-based strategies to increase WIC retention and redemption rates. J Hunger Environ Nut 17, 460–474. doi: 10.1080/19320248.2021.1915906.

